# The development of an online database for interventions tested in transgenic mouse models of Alzheimer's disease

**DOI:** 10.1002/ebm2.10

**Published:** 2015-08-11

**Authors:** K. J. Egan, H. M. Vesterinen, S. K. McCann, E. S. Sena, M. R. MacLeod

**Affiliations:** ^1^ Department of Clinical NeurosciencesUniversity of EdinburghEdinburghUK; ^2^ Stroke DivisionFlorey Institute of Neuroscience and Mental HealthMelbourneAustralia

**Keywords:** data, transgenic mouse models, Alzheimer's disease, translational failure

## Abstract

Despite many efforts by the research community, Alzheimer's disease (AD) is still an incurable neurodegenerative condition that affects an estimated 44 million individuals worldwide and this figure is expected to increase to 135 million by the year 2050. As the research community currently reflects on previous endeavours, it is essential that we maximize the use of existing knowledge to inform future trials in the field. This article describes the development of a systematically identified data set relating to over 300 interventions tested in over 10,000 animals. The data set includes cohort‐level information for six structural outcomes and six behavioural assessments. We encourage others to use this dataset to inform the design of future animal experiments modelling AD and to promote effective translation to human health.

## Introduction

Alzheimer's disease (AD) is an incurable neurodegenerative condition that affects an estimated 44 million individuals worldwide. This figure is expected to increase to 135 million by the year 2050,^1^ placing increasing social and economic strain on society in the years ahead.^2^ The societal costs of AD have been estimated to be US$604 billion per year,^3^ with the burden of care commonly falling on the shoulders of family members, impacting on communities and society as a whole.

Currently, symptomatic relief can be achieved for some patients using acetylcholinesterase inhibitors or memantine, but these are not suitable or effective option for all patients at all stages. There is therefore a pressing need both for interventions capable of providing greater symptomatic relief, and for disease‐modifying interventions therapies that might slow, or halt or even reverse, the progression of the condition.

Substantial efforts have been made in preclinical science in order to identify candidate clinical treatments. Much focus for disease‐modifying therapy has concentrated on targets identified by the amyloid hypothesis including active and passive immunization strategies (e.g. AN‐179, Bapineuzumab) and gamma secretase inhibitors (e.g. Semagacestat, Tarenflurbil and Avagecestat).^4^, ^5^

Following the identification of familial AD and those mutated genes responsible, transgenic mouse models have been designed to recapture aspects of AD *in vivo* for two decades. The first transgenic mouse model was engineered in 1995^6^ where the overexpression of a mutated APP (V717F mutation driven by the platelet‐derived growth factor promoter) produced a phenotype of amyloid plaques and neuronal loss. Subsequently, there has been an array of models produced, including models based on the expression of transgenic presenilin (PS)^7^; or both APP and PS each with specific AD like pathologies alongside behavioural deficits.^8^ Collectively, such work culminated in crossing APP, PS and Tau lines to produce a tripe transgenic model (3xTgAD), capable of capturing; tau neurofibrillary tangles, aggressive amyloid plaques and cognitive deficits.^9^ The success regarding the development of transgenic mouse models has encouraged extensive testing of candidate intervention strategies before reaching the clinical trial stage.^10^ Both behavioural end points (e.g. paradigms such as the Morris water maze^11^) and pathological end points (e.g. enzyme‐linked immunosorbent assay for amyloid beta levels, immunohistochemistry for plaques) can be quantified and efficacy determined by comparing control and treatment groups.

The prospect of testing candidate intervention strategies in animal models capable of capturing aspects of AD provides opportunities both to demonstrate efficacy *in vivo* and to investigate molecular mechanisms. While transgenic mouse models have certainly advanced our understanding of AD, their utility in developing new treatments has been less certain; Zahs and Ashe^10^ showed that while over 300 interventions had been tested in the Tg2576 mouse, none had met with clinical success. Indeed, no novel clinical treatments have emerged in AD despite over a decade of testing therapeutics in these transgenic animals.

There are many plausible reasons for translational failure in AD,^12^ one of which is that animal transgenic studies have overestimated the reported efficacy. If this were true, it would be essential that we understand better the internal validity (e.g. sample size calculations, blinding and randomization) and external validity (e.g. are we testing interventions in conditions representative of the clinical setting) of such experiments. The impact of these factors in a given field of research can be identified through systematic review, and these findings disseminated to the wider scientific community to inform improvements in research practice. Subsequent meta‐analysis can also be performed, with the caveat that however systematically constructed a data set is unlikely to include all experimental data; some experiments will have been published after the search was performed and there is also likely to be at least some publication bias and selective outcome reporting bias. Nevertheless, by making this resource available to the community we hope to empower AD researchers by allowing to set their research plans and research findings in the context of what is already known.

Here we have conducted a systematic review of transgenic mouse studies testing the efficacy of candidate drugs in transgenic models of AD. We have extracted data from these studies for meta‐analysis, the results of which will be presented elsewhere. To allow others to benefit from these data we are now making these available to the scientific community as a whole. Of the many possible uses, we consider this data set will be of most immediate use to others planning preclinical AD studies (particularly to inform sample size calculations) or clinical trials of candidate drugs included in this review. Those interested in using these data to empirically guide future research can find further guidance on the analyses of datasets such as these in Vesterinen et al.^13^ and an example of the specific application of these techniques to RCT design can be found in a description of the MS SMART drug repurposing trial in multiple sclerosis.^14^ We envisage that the development of machine learning text mining tools will allow this database to be updated in the future, and as those tools evolve it may be possible for such databases to be updated in real time.

## Methods

Studies in animal models of AD were identified from 1995 to Jan 2009 in Pubmed, EMBASE and ISI Web of knowledge with the search terms [“targeted deletion” OR “overexpression” OR “knock out” OR “vector” OR “transgenic”] AND [“dementia” OR “tau” OR “mild cognitive impairment” OR “Alzheimer's disease”] with the search limited to animals using the filters available in those databases. The searches were taken from 1995 as this year coincides with the production of the first transgenic AD mouse model. The search was conducted in January 2009. The protocol for this systematic review was defined in advance, and was amended in Feb 2011 (hierarchy for deciding which cohort to be used as a control group) and March 2011 (definition of method of calculating area under the curve for Morris Water Maze acquisition phase).

## Inclusion Criteria

Publications were included that tested interventions using any amyloid‐, tau‐ or PS‐based transgenic mouse model of AD.

## Exclusion Criteria

Genetic manipulations surplus to those required to produce symptoms of the condition were excluded as with genetic treatments if intervention took place before birth (i.e. gene knock‐outs or knock‐ins). We excluded studies where more than one treatment was administered. For the MWM, reversal task behaviour nor time in opposite or adjacent quadrants were extracted.

## Quality Score

Publications identified were assessed against a five‐point study quality checklist adapted from those previously described in the “Good Laboratory Practice Guidelines” for stroke modelling.^15^ These items included a statement regarding (1) random allocation to group, (2) blinded assessment of outcome, (3) sample size calculation, (4) compliance with animal welfare legislation and (5) declaring a conflict of interest. One point was awarded for each criterion reported.

## Results

Our systematic search identified 427 publications testing interventions in transgenic mouse models of AD. These publications described 357 interventions and 55 transgenic models representing 11,118 animals and 838 experiments. All extracted data are available through Figshare (see *Referencing* section for web address).

**Figure 1:**
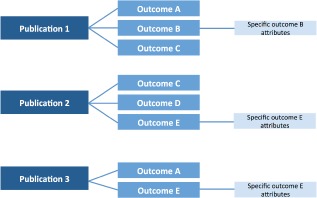
Overall structure of recorded information. Information was organized across publication level and outcomes with a one to many relationship. For selected outcomes, further information was also extracted regarding the methodology of the individual experiments (see later).

**Figure 2:**
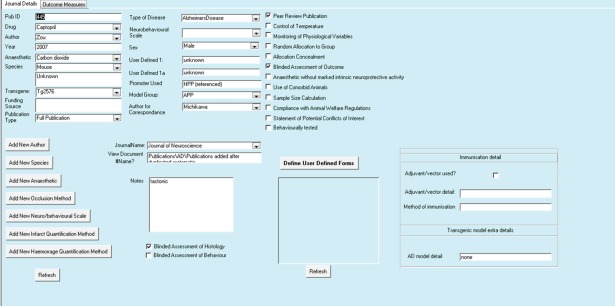
Screenshot of publication level entry form. This form is used to record information regarding the publication as a whole. Information included specifically relates to details of the author, year or overall methodological approaches and study quality items. See Table 1 for details.

**Figure 3:**
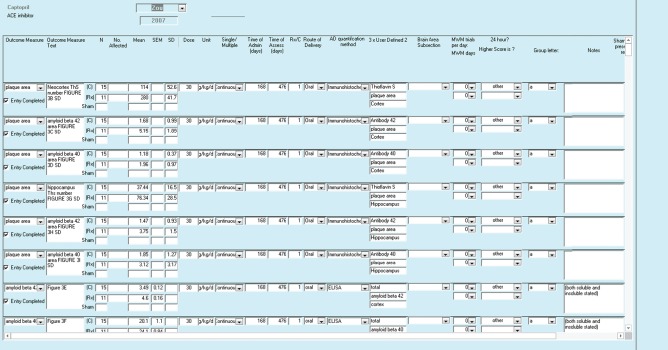
Screenshot of outcome level entry form. This form is used to record information variables essential to meta‐analysis including the mean variance and number of animals in each group. Alongside these details are data concerning the specific methodology of the experiment including the drug, dose, dose units, route of drug administration and details regarding the age at which interventions are administered and outcomes are assessed. See Table 2 for details.

We organized extracted data into a hierarchy of three principal areas: publication level details, outcome level details and additional specific attributes of individual outcomes (see Figure 1). For publication level details, see Table 1 and Figure 2. For Outcome level details, see Tables 2 and 3 and Figure 3.

**Table 1:** The organization of publication level information. These 12 field codes (A1 to A12) are used to record information regarding the publication as a whole such as information specifically regarding the author or overall methodological approaches and study quality items. See text for more details

Item	Field code	Description	Notes
Unique ID	A1	Primary key	Automatically generated number
Year	A2	Year of publication	Numerical entry
Author	A3	Primary author	Text (lookup table)
Anaesthetic used	A4	Anaesthetic used at the time of sacrifice	Text (lookup table)
Background strain	A5	Detail regarding the background strain of the mice	Text (lookup table)
Transgene	A6	Specific transgenic mutations, or knock‐out used	Text (lookup table)
Type of publication	A7	Description of whether publication is a full publication or an abstract	Publication/abstract
Blinded assessment of outcome	A8	Reporting in publication that studies were blinded	Y/N
Random allocation to group	A9	Reporting in publication that animals were randomly allocated to treatment groups	Y/N
Sample size calculation	A10	Reporting in publication of how the sample size of the study population was statistically identified	Y/N
Compliance with animal welfare legislation	A11	Reporting of compliance with any animal welfare legislation	Y/N
Statement regarding potential conflicts of interest	A12	Reporting of a conflict of interest statement	Y/N

**Table 2:** The organization of outcome level information. These 22 field codes (B1 to B22) are used to record more specific information regarding individual experiments. These data include variables essential to meta‐analysis including the mean variance and number of animals in each group. Alongside these details are data concerning the specific methodology of the experiment including the drug, dose, dose units, route of drug administration and details regarding the age at which interventions are administered and outcomes are assessed

Item	Field code	Description	Notes
Outcome measure	B1	Specific outcome measure of interest (e.g. plaque pathology, tau or neurodegeneration)	Text (lookup table)
Publication ID	B2	Unique primary key for each study within a given publication (e.g. each drug examined within a publication would be assigned a new publication ID)	Automatically generated number
Outcome measure ID	B3	Unique primary key for individual outcomes	Automatically generated number
Group letter	B4	Unique primary key for individual cohorts. Combined with publication ID identifies where specific cohorts exists	Text (lookup table)
Number of animals in control group	B5	Most conservative estimate of control animals used	Numerical entry
Number of animals in treatment group	B6	Most conservative estimate of treatment animals used	Numerical entry
Number of wild type animals	B7	Most conservative estimate of wild type animals used	Numerical entry
Mean in control group	B8	Mean in control group	Numerical entry
Variance in control group	B9	Variance in control group (standard deviation or standard error of the mean)	Numerical entry
Mean in treatment group	B10	Mean in treatment group	Numerical entry
Variance in treatment group	B11	Variance in treatment group (standard deviation or standard error of the mean)	Numerical entry
Mean in wild type group	B12	Mean in wild type group	Numerical entry
Variance in wild type group	B13	Variance in wild type group (standard deviation or standard error of the mean)	Numerical entry
Age at intervention administration	B14	Age in days of animals at the time of first intervention administration	Numerical entry
Age at outcome assessment	B15	Age in days of animals at the time of outcome assessment	Numerical entry
Dose	B16	Drug dose used	Numerical entry
Dose units	B17	Drug dose units	Numerical entry
Route of drug delivery	B18	Route of drug into body	Text (lookup table)
Number of treatment groups per control	B19	How many time a control group serves in study	Numerical entry
Drug	B20	Intervention tested	Text (lookup table)
Sex	B21	Sex of animal used	Text (lookup table)
Animal type	B22	Type of animal used	Text (lookup table)

**Table 3:** The organization of specific outcome attributes information. These field codes (C1 to C12) provide further context on the details regarding individual techniques or experiments. For example, for behavioural experiments that use the Morris water maze, specific information regarding the methodological set up can be useful to experimenters for planning future experiments (see C1 to C5)

Outcome and item	Field code	Description	Notes
**Morris water maze**			
Size of pool	C1	Diameter of the pool used in metres	Numerical entry
Water temperature	C2	Average temperature of the water used in degrees Celsius	Numerical entry
Number of days training	C3	Number of total days training (can be different from acquisition curve points)	Numerical entry
Training sessions per day	C4	Number of acquisition training sessions per day	Numerical entry
Time point (acquisition only)	C5	Specific time point number during acquisition training	Numerical entry
**Plaque burden**			
Staining technique used	C6	Description of whether plaques are stained with immunohistochemical methods, congo red or Thioflavin S	Text (lookup table)
**Amyloid beta 40/42**			
Solubility	C7	Soluble, insoluble or total amyloid measured	Text (lookup table)
**Tau**			
Description of tau entity	C8	Description of whether outcome represents “overall” tau or “phosphorylation state” of tau	Text (lookup table)
Measurement technique	C9	Description of tau antibody used	Text (lookup table)
**Cellular infiltrates**			
Data type	C10	Details of whether data astrocytes or microglia.	Text (lookup table)
**Neurodegeneration**			
Outcome type	C11	Description of whether neurodegeneration outcome might be considered a "direct" or "indirect" measure	Text (lookup table)
Outcome measure specificity	C12	Specific methodology used to assess neurodegeneration	Text (lookup table)

### outcome level details

For experimental outcome measures, we extracted over 4,000 individual outcome measure comparisons where a control group was compared to that of the treatment group. We connected these to the publication level using a one‐to‐many relationship and thus could record multiple outcomes for individual studies. Structural outcomes were particularly prevalent with publications three times more likely to report this compared to behavioural outcomes.

For some structural outcomes, it was of interest to extract further information than the basic outcome measure level data. For example, for plaque burden there were three commonly used staining techniques used: immunohistochemistry, congo red and Thioflavin S and such details were recorded attached to the outcome measure level.

For cellular infiltrates, we recorded whether outcomes represented astrocytosis or microgliosis and for tau we recorded whether data represented phospho tau (and specific phosphorylation site) or overall tau levels. Likewise, we extracted information regarding neurodegeneration according to the specific variable measured.

### neurobehavioural outcomes

We identified six key neurobehavioral outcome measures; the training (acquisition phase) and the test (probe phase) of the Morris water maze, Radial arm water maze, fear conditioning, the Y maze and T maze.

For the Morris water maze, we were able to extract a number of paradigm‐specific methodological attributes. For example, we extracted data recording the number of days training, the number of training sessions per day, the temperature of the water in the Morris water maze, the diameter of the pool and the time delay between the end of the acquisition phase and the probe “test” phase assessment. In addition, for acquisition time points we measured all data points and associated errors.

## Referencing

Other investigators are welcome to use, with attribution, the data reposited on Figshare http://plos.figshare.com/articles/Interventions_tested_in_preclinical_studies_using_transgenic_mouse_models_of_AD/1185428. This should be referenced through reference to this publication.

## Conflict of Interest

The authors declare that there are no conflicts of interest.
